# Supplementary material for: Low rate of SARS-CoV-2 incident infection identified by weekly screening PCR in a prospective year-long cohort study
Source: PLoS One. 2022 Sep 26;17(9):e0274078. doi: 10.1371/journal.pone.0274078 (PMC9512209; doi:10.1371/journal.pone.0274078)
Supplement: Attachment — Submitted filename: SARS2_Prospective_Epi_ReviewerResponse_8Aug22.doc [file pone.0274078.s002.doc]

**Editorial Comments:**

1. Please ensure that your manuscript meets PLOS ONE's style requirements, including those for file naming. The PLOS ONE style templates can be found at 
[https://journals.plos.org/plosone/s/file?id=wjVg/PLOSOne_formatting_sample_main_body.pdf](https://urldefense.com/v3/__https:/journals.plos.org/plosone/s/file?id=wjVg*PLOSOne_formatting_sample_main_body.pdf__;Lw!!NuzbfyPwt6ZyPHQ!vd05jkYLgl9TttiqBVVZGnpLQ28Ci8Paz3V61ofcseeRVvU617U2JSZxn3D84pTDENuDOOFf9-bAB8MPgWLSsgyQEVo$) and 
[https://journals.plos.org/plosone/s/file?id=ba62/PLOSOne_formatting_sample_title_authors_affiliations.pdf](https://urldefense.com/v3/__https:/journals.plos.org/plosone/s/file?id=ba62*PLOSOne_formatting_sample_title_authors_affiliations.pdf__;Lw!!NuzbfyPwt6ZyPHQ!vd05jkYLgl9TttiqBVVZGnpLQ28Ci8Paz3V61ofcseeRVvU617U2JSZxn3D84pTDENuDOOFf9-bAB8MPgWLSZR54zgU$)

We have edited the manuscript to meet PLOS ONE style requirements throughout.

2. You indicated that you had ethical approval for your study. In your Methods section, please ensure you have also stated whether you obtained consent from parents or guardians of the minors included in the study or whether the research ethics committee or IRB specifically waived the need for their consent.

We have clarified that guardians provided written consent for all children and that children >= 7 also provided verbal assent.

3. We note that the grant information you provided in the ‘Funding Information’ and ‘Financial Disclosure’ sections do not match.

When you resubmit, please ensure that you provide the correct grant numbers for the awards you received for your study in the ‘Funding Information’ section.

Grant numbers and funding institutions have been corrected in the ‘Funding Information’ section.

**Reviewers' Comments:**

Reviewer #1:

The manuscript submitted by Frenkel et al and entitled “Low rate of SARS-CoV-2 incident infection identified by weekly screening PCR in a prospective year-long cohort study” evaluates the utility of asymptomatic screening for SARS-CoV-2 in low-risk populations. In this work 553 seronegative individuals were followed for 1 year and tested using self-collected nasal swabs for rtPCR testing. Of the subjects tested, only 9 cases were identified as positive and included subjects at greater risk, HCP, house member of a HCP, student, and daycare case. Furthermore, due to the once-a-week screening, 1 of 9 were identified prior to symptom onset and self-requested testing. Overall, the study is well written and demonstrates that in low risk, high educated settings, a weekly screening had low impact on identifying pre-symptomatic individuals. A few modifications should be addressed prior to publication.

Major Comments
What was the prevalence in the area during the period of testing and do you have that available for asymp vs symptomatic subjects (greater area vs study population).

We thank the reviewer for their feedback. The prevalence of symptomatic infection in the greater area (King County) varied across the year but reached a peak 7-day incidence of more than 200 cases / 100,000 population. This information is presented in the fourth paragraph of the discussion, along with a citation to the King County Department of Health website with full rolling incidence of infection throughout the pandemic. Unfortunately, to the best of our knowledge there were no large studies of asymptomatic individuals from the larger population during the time period of our study, so this information is not available for comparison.

I would also suggest adding the date range to the materials and methods section.

We have moved the information on date range of enrollment from the first sentence of the Results to the first sentence of the Material and Methods.

Minor Comments
Ln 66: Why was transport media not used for the study and was there any stability testing done in house to demonstrate that dry swabs remained detectable over the 72 hours.

At the time of the study there were transport media shortages globally, so we conducted in house testing to demonstrate that dry swabs retained similar sensitivity up to 72 hours. Specifically, we compared the cycle threshold (Ct) values of nasal swabs spiked with 50ul of a SARS-CoV-2 positive specimen collected in universal transport medium (UTM), when held in UTM, PBS, or dry, and found that the cycle time remained stable over 72 hours regardless of storage condition. A comment noting this is now included in the Materials & Methods.

Figure 1: What do the 2 no stars represent? Is that no specific variable determined?

We have clarified in the figure legend that “no stars” represents the two cases with no risk factor for infection identified.

Conclusion: Add a limitation that contacts are all self-reported and as this was work done at a place of employment, individuals may have misrepresented their social interactions.

We have added these limitations to the conclusions.

Reviewer #2:

This is a very interesting study providing intriguing evidence for the usefulness of the weekly testing with rtPCR. RTPCR has its limits due to the price and the need to purify the samples. In one recent study (PMID: 35395856), completely alternative approach was suggested with much faster turnaround time without compromising the quality, sensitivity and specificity. Maybe authors can elaborate the discussion about this point a bit more. Would the faster and cheaper testing method improve the proposed population surveillance outcomes?

We appreciate the reviewer calling this publication to our attention. We have added a brief discussion of how alternative testing approaches may change the value of surveillance to the manuscript, citing this publication.

Reviewer #3: This is a very nice paper.

Short Title: I think that shortening SARS-CoV-2 to SARS2 is unwise. Perhaps “rate of incident” can be changed to “incidence” to accommodate more complete nomenclature.

We appreciate this feedback and have edited the Short Title to: “Low incidence of SARS-CoV-2 by weekly PCR”

Line 76: How was the sensitivity of detection for the nasal specimens determined?

The sensitivity of detection was determined using 2-fold serial dilutions of an in-vitro transcribed SARS-CoV-2 RNA control spiked into SARS-CoV-2 negative nasal swab matrix (nasal swabs collected from healthy donors). The analytical sensitivity of the diagnostic RT-qPCR was estimated at 10 SARS-CoV-2 RNA copies/reaction (equivalent to 250 copies of SARS-CoV-2 per nasal swab eluted in 1 mL of PBS) utilizing a cycle threshold cut-off of 40. To confirm the limit of detection (LoD) we tested 20 replicate samples at 1x and 20 replicates at 2x the estimated LoD, resulting in 95% positive replicates at 250 copies/swab and 100% positivity at 500 copies/swab. We have included a brief description of our approach in the third paragraph of the Materials & Methods.

I think that it should be possible to address the HIPAA considerations in creating a deidentified public data set by replacing dates with the intervals between dates, and use of age groupings rather than age. This should represent sufficient deidentification. The questionnaire data obviously constitutes identification, but the details are unimportant for purposes of this paper.

We appreciate this suggestion and have generated a primary data set with age blocks as suggested and vaccine dates removed, but compiled number of vaccinated individuals per week of the study presented.
